# Interactions Controlling the Slow Dynamic Conformational Motions of Ubiquitin

**DOI:** 10.3390/molecules22091414

**Published:** 2017-08-28

**Authors:** Soichiro Kitazawa, Maho Yagi-Utsumi, Koichi Kato, Ryo Kitahara

**Affiliations:** 1 College of Pharmaceutical Sciences, Ritsumeikan University, Noji-higashi 1-1-1, Kusatsu 525-8577, Japan; skitaza@fc.ritsumei.ac.jp; 2 Okazaki Institute for Integrative Bioscience and Institute for Molecular Science, National Institutes of Natural Sciences, Myodaiji-cho, Aza-higashiyama 5-1, Okazaki 444-8787, Japan; mahoyagi@ims.ac.jp (M.Y.-U.); kkatonmr@ims.ac.jp (K.K.); 3 Graduate School of Pharmaceutical Sciences, Nagoya City University, Tanabedouri 3-1, Mizuho-ku, Nagoya 467-8603, Japan

**Keywords:** alternatively folded state, high-pressure NMR, ubiquitin

## Abstract

Rational mutation of proteins based on their structural and dynamic characteristics is a useful strategy for amplifying specific fluctuations in proteins. Here, we show the effects of mutation on the conformational fluctuations and thermodynamic stability of ubiquitin. In particular, we focus on the salt bridge between K11 and E34 and the hydrogen bond between I36 and Q41, which are predicted to control the fluctuation between the basic folded state, N_1_, and the alternatively folded state, N_2_, of the protein, using high-pressure NMR spectroscopy. The E34A mutation, which disrupts the salt bridge, did not alter picosecond–to–nanosecond, microsecond–to–millisecond dynamic motions, and stability of the protein, while the Q41N mutation, which destabilizes the hydrogen bond, specifically amplified the N_1_–N_2_ conformational fluctuation and decreased stability. Based on the observed thermodynamic stabilities of the various conformational states, we showed that in the Q41N mutant, the N_1_ state is more significantly destabilized than the N_2_ state, resulting in an increase in the relative population of N_2_. Identifying the interactions controlling specific motions of a protein will facilitate molecular design to achieve functional dynamics beyond native state dynamics.

## 1. Introduction

Proteins in solution exist in thermodynamic equilibrium and fluctuate between multiple conformational states, from folded to unfolded. Increasing evidence shows that conformations with higher Gibbs free energy than the basic folded state (the native state) are important for functions such as enzymatic reactions [1,2] and signal transduction [3]. However, the structure, dynamics, and stability of high-energy states of proteins are not as well understood as those of the basic folded state. In fact, little is known about how functional motions are regulated in protein molecules.

Ubiquitin, which consists of 76 amino acid residues, is conjugated to target proteins by a specific E1–E2–E3 cascade reaction and plays essential roles in multiple cellular functions in eukaryotes [4,5]. The three-dimensional structure of ubiquitin has been studied in crystal structures by X-ray diffraction [6] and in solution by nuclear magnetic resonance (NMR) spectroscopy [7], both of which have revealed an essentially identical structure of the protein. Based on studies of the structure of ubiquitin in complex with other proteins, such as E1 and E2 enzymes and ubiquitin-associated proteins [8,9,10,11], ubiquitin uses different interaction modes for specific protein targets. Moreover, NMR studies have revealed that, in solution, ubiquitin undergoes a variety of conformational fluctuations within and beyond the native state ensemble [10,12,13,14,15,16].

The previous high-pressure NMR studies have revealed the presence of two high-energy states of wild-type ubiquitin, between the basic folded and totally unfolded states, namely the alternatively folded state, N_2_, and the locally disordered state, I [17,18,19]. Nuclear Overhauser Effect (NOE)-based structural determination by high-pressure NMR spectroscopy showed reorientation of the α-helix and β_5_-strand at 3 kbar, where 78% of the protein molecules were in the N_2_ state, and corresponding changes in several intra-molecular interactions [18]. The structural changes were also supported by residual dipolar coupling (RDC) experiments [20] and molecular dynamics (MD) simulations [21] of the protein under high pressure. 

Based on the structural characteristics of N_2_, we constructed ubiquitin mutants, which, we hypothesized, would stabilize the N_2_ state [22]. In particular, we focused on two interactions that are predicted to control the N_1_–N_2_ fluctuation by high-pressure NMR studies. The first is the salt bridge between K11 and E34, which joins the α-helix and the β_2_-strand; the second is the hydrogen bond between I36 and Q41, which is widely conserved in ubiquitin-family proteins, such as ubiquitin and NEDD8 [11]. For instance, in the Q41N mutant, N_2_ was 70% populated at atmospheric pressure [22] and 97% populated at 2.5 kbar [23]. Surprisingly, in the Q41N mutant at 2.5 kbar, N_2_ has a very similar conformation to that of ubiquitin in complex with ubiquitin-activating enzyme E1 [23]. Moreover, the high-energy states, N_2_ and I, are conserved in ubiquitin–like modifiers, such as the NEDD8 [24] and SUMO-2 [25] proteins, and NEDD8 shows a similar reorientation of the β_5_-strand when it binds to NEDD8-activating enzyme E1 [24]. Based on these results, we hypothesized that the N_1_–N_2_ fluctuation is a common molecular phenomenon for the E1 recognition of ubiquitin-like modifiers [23].

In the present study, we further investigated the effects of mutations in the residues involved in the hydrogen bond and salt-bridge on backbone dynamics and stability, and reveal how the N_2_ state is stabilized in each mutant.

## 2. Results and Discussion

### 2.1. Chemical Shift Changes Induced by Mutations

**Figure 1:**
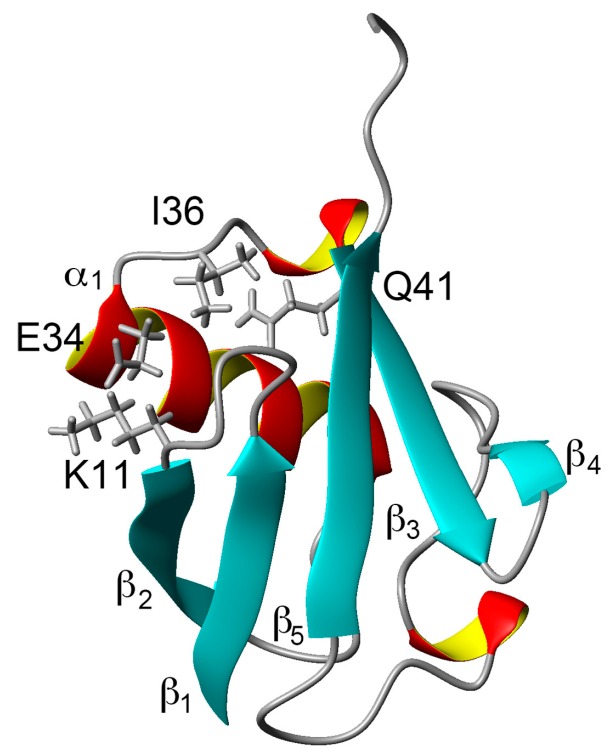
Three-dimensional structure of ubiquitin (PDB ID: 1D3Z). Sidechains of K11, E34, I36, and Q41 are shown as stick models. α-helices are marked by red and yellow, β-sheets are marked by cyan.

**Figure 2:**
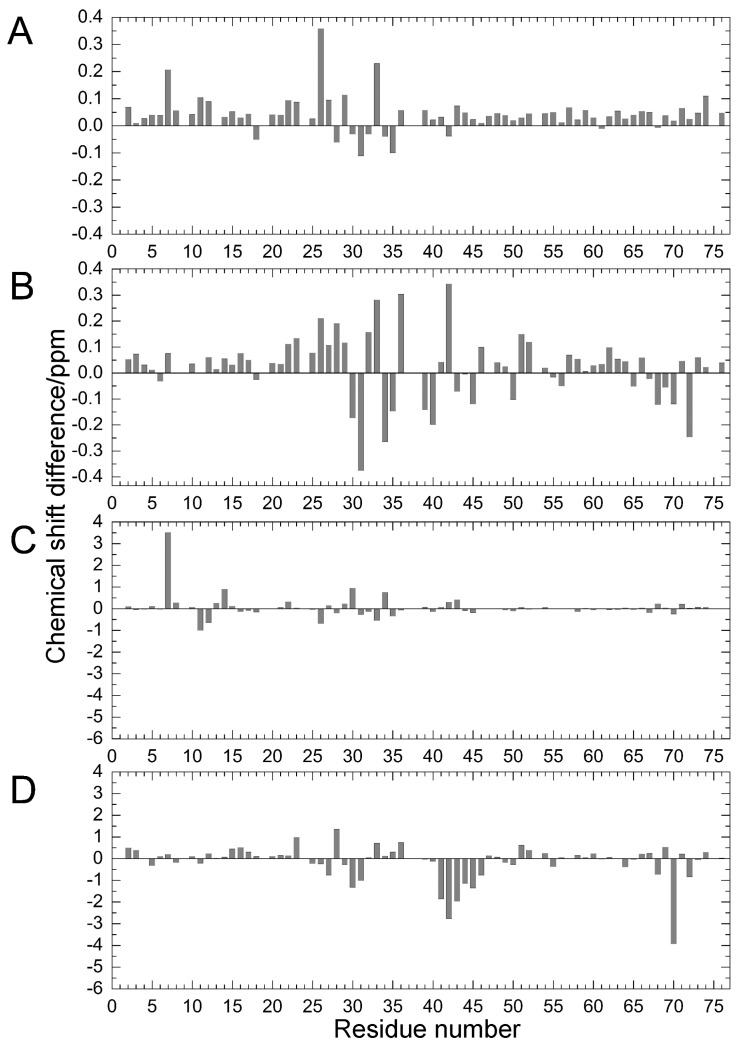
Chemical shift differences (Δ*δ*/ppm) between mutants and WT proteins: (**A**) amide ^1^H for E34A; (**B**) amide ^1^H for Q41N; (**C**) amide ^15^N for E34A; and (**D**) amide ^15^N for Q41N. Data for Q41N (panels **B** and **D**) were obtained from the literature [22] for comparison (Reproduced with permission from American Chemical Society).

Comparing the structural characteristics of N_1_ and N_2_, we focused on the interactions of the salt bridge between K11 and E34, which ties the α–helix to the β_2_-strand, and the hydrogen bond between I36 and Q41, which ties the loop (residues 35–40) to the β_3_-strand (Figure 1). It is noteworthy that this hydrogen bond is highly conserved among proteins in the ubiquitin family [11]. We previously created K11A, E34A, Q41A, and Q41N mutants to disrupt or reduce these interactions and collected ^1^H/^15^N HSQC spectra (Figure 1 in [22]). Figure 2 shows ^1^H and ^15^N chemical shifts of the backbone amide groups in the E34A and Q41N mutants alongside the residue number. The changes in the ^1^H and ^15^N chemical shifts for Q41N were much larger than those for E34A. In the case of Q41N, large chemical shift changes were observed not only near the substituted site but also at residues 25–45 and 68–72, which match the residues at which changes are observed when the WT protein is under high pressure [18]. As previously noted, the chemical shift changes observed in the Q41N mutant, which showed the greatest chemical shift changes among the mutants, were significantly correlated with those induced by pressure perturbation [22]. This indicated that the N_2_ state is stabilized by the Q41N mutation, similar to the WT protein under pressure. Because the magnitude of the chemical shift changes was much larger in the Q41A and Q41N mutants than in the K11A and E34A mutants, the hydrogen bond between I36 and Q41 appears to be more important for controlling the N_1_–N_2_ conformational fluctuations of the protein (Figure 1 in [22]).

### 2.2. Stability of WT and Mutant Proteins

**Figure 3:**
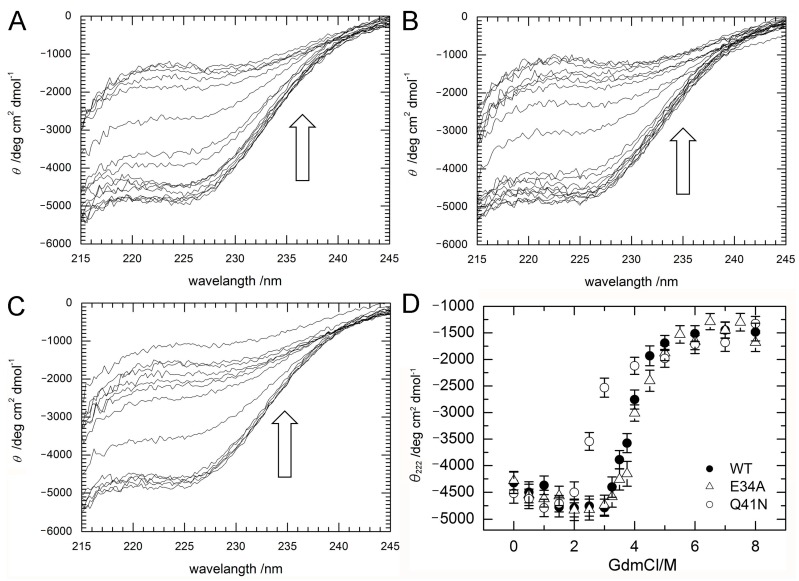
(**A**–**C**) CD spectra (**A**; WT; **B**; E34A, **C**; Q41N) and (**D**) molar ellipticity, *θ*_222_ of WT (closed circles) and mutant (E34A; open triangles and Q41N; open circles) ubiquitin as a function of guanidine hydrochloride concentration. CD spectral changes by increasing a concentration of GdmCl are indicated by arrows (⇧). Molar ellipticity was calculated from CD absorption at 222 nm (see Materials and Methods). Error-bars show root mean square deviation from the average.

To investigate stability changes induced by the mutations, the effects of guanidine hydrochloride (GdmCl) on the denaturation of ubiquitin WT, E34A, and Q41N were examined at 298 K. Circular dichroism (CD) spectra, namely the molar ellipticity, *θ*_obs_, calculated from the CD absorbance at 215–245 nm, of the proteins at various concentrations of GdmCl were shown in panels A–C of Figure 3. With increasing a concentration of GdmCl, *θ*_obs_ at 215–235 nm wavelength of the proteins were slightly increased (downside) up to 2 M and then sharply decreased (upside).

Figure 3D shows changes in the molar ellipticity at a wavelength of 222 nm, *θ*_222_, which is a major index of the secondary structure of proteins, as a function of GdmCl concentration. The curves represent averaged transitions from the folded states (N_1_ and N_2_) to the unfolded state (U). *θ*_obs_ can be expressed by Equation (1) (see Material and Methods). The Δ*G* values for unfolding obtained by curve fitting were 33 ± 8 kJ/mol for WT, 27 ± 7 kJ/mol for E34A, and 17 ± 5 kJ/mol for Q41N. The *m*-values for unfolding were 8 ± 2 kJ/(mol M) for WT, 7 ± 2 kJ/(mol M) for E34A, and 6 ± 2 kJ/(mol M) for Q41N. Unlike E34A, Q41N showed significantly lower thermodynamic stability than the WT protein. These results indicate that, although the K11 and E34 side chains are located at positions that enable them to form a salt bridge in the crystals [6], the contribution of the salt bridge between K11 and E34 to protein stability in solution is not as significant as that of the hydrogen bond between I36 and Q41. In addition, the smaller *m*-value for Q41N than that for WT also may indicate that the folded conformation of Q41N has more open and hydrated surfaces, than that of WT.

### 2.3. pK_a_ Values for Side Chains of Aspartate and Glutamate 

**Figure 4:**
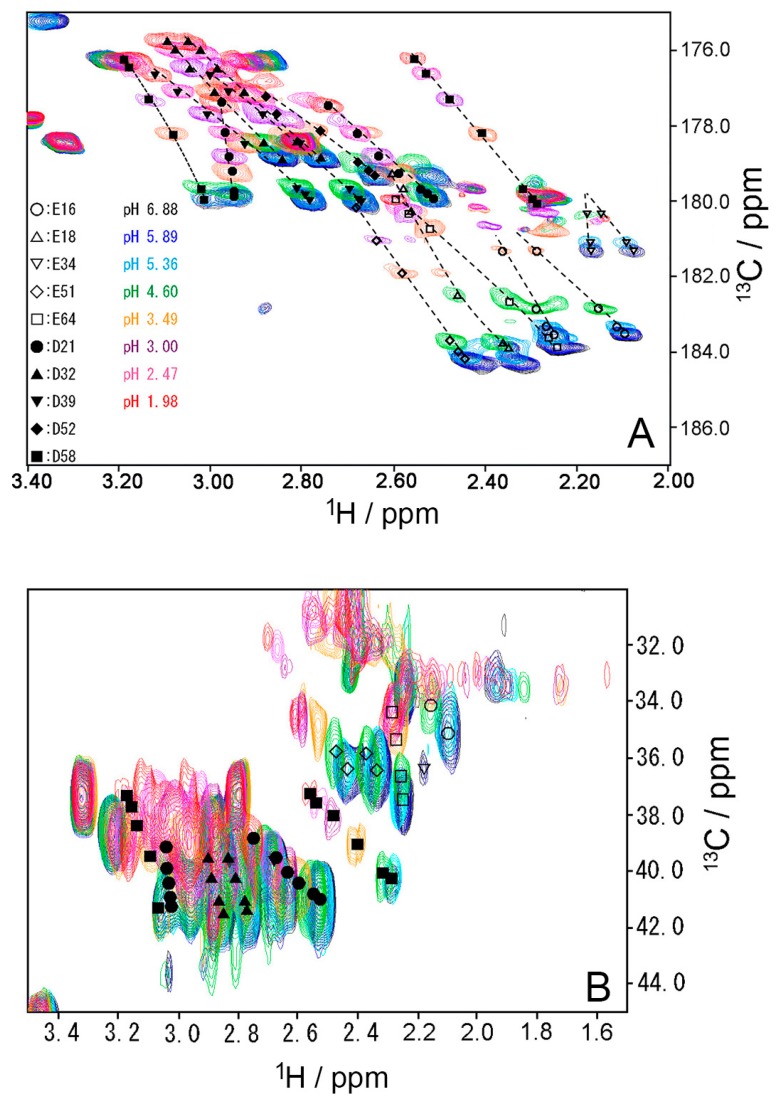
(**A**) Two-dimensional H (C) CO and (**B**) HC (CO) spectra of WT ubiquitin at representative pH conditions from 6.88 (black) to 1.98 (red) at 298 K. All peaks move up the top, when pH decreases.

**Figure 5:**
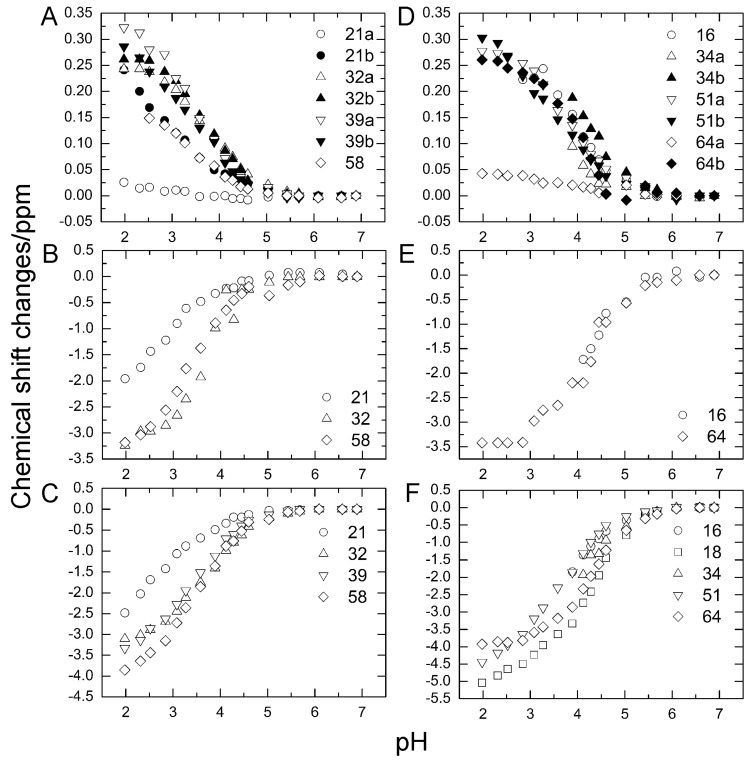
Changes in aspartate (**A**–**C**) and glutamate (**D**–**F**) chemical shifts of WT ubiquitin by pH titration from pH 6.88 to pH 1.98 at 298 K. (**A**) Hβ of aspartate; (**B**) Cβ of aspartate; (**C**) C′ of aspartate, (**D**) Hβ of glutamate; (**E**) Cγ of glutamate; (**F**) C′ of glutamate.

To investigate the stability of the salt bridges in solution, we determined the p*K*_a_ values for the aspartate and glutamate side chains using NMR spectroscopy. We performed two-dimensional H (C) CO and HC (CO) experiments for the WT protein at 18 different pH values from 1.98 to 6.88. The H (C) CO experiment revealed correlations between Hβ/Hγ and C′ in these side chains, and the HC (CO) experiment revealed a similar correlation between Hβ/Hγ and Cβ/Cγ; thus, they provided chemical shift values for the nuclei [26]. Figure 4 shows the H (C) CO spectra (panel A) and the HC (CO) spectra (panel B) at the different pHs. Figure 5 shows the pH titration curves for Hβ/Hγ (panels A and D), Cβ/Cγ (panels B and E), and C′ (panels C and F) for aspartate (panels A–C) and glutamate (panels D–F). Table 1 lists the p*K*_a_ values for the aspartate and glutamate side chains determined by the global fitting of the observed chemical shift data to a Henderson–Hasselbalch equation [27,28]. Most of the transition curves with single p*K*_a_ values could be satisfactorily fitted to the NMR data. The p*K*_a_ values of the residues, except E34 and E64, were in accord with published data that had been estimated using ^1^H chemical shifts only, in a range of ±0.2 pH units [27]. With the exception of the D21 and E51 side chains, most of the side chains of aspartate and glutamate showed p*K*_a_ values similar to those of model compounds for side-chain carboxyl groups in oligopeptides (3.8–4.1 for aspartate and 4.1–4.6 for glutamate) [28]. Although the relationships between carboxyl p*K*_a_ values and protein structure are not very precise [28], the present results suggest that the carboxyl groups, including those of E34, are exposed to the solvent and do not significantly interact with their counterparts under the conditions of the solution in our study (i.e., 20 mM d–Tris HCl buffer at pH 7.2). This is consistent with the fact that the salt bridge between K11 and E34 does not significantly contribute to protein stability. It should be noted that the presence of ions in buffer agents and fluctuations of polypeptide chains, however, may reduce the stability of salt bridges in solution.

In contrast, the p*K*_a_ values of the D21 and E51 carboxyl groups were much lower than those of the model compounds. Considering the three-dimensional structure of the protein, the D21 and E51 carboxyl groups are expected to interact with the K29 and R54 side chains, respectively. However, because these residues are not involved in the N_1_–N_2_ conformational fluctuation, these interactions were not investigated further.

### 2.4. Backbone Dynamics and Hydration

**Figure 6:**
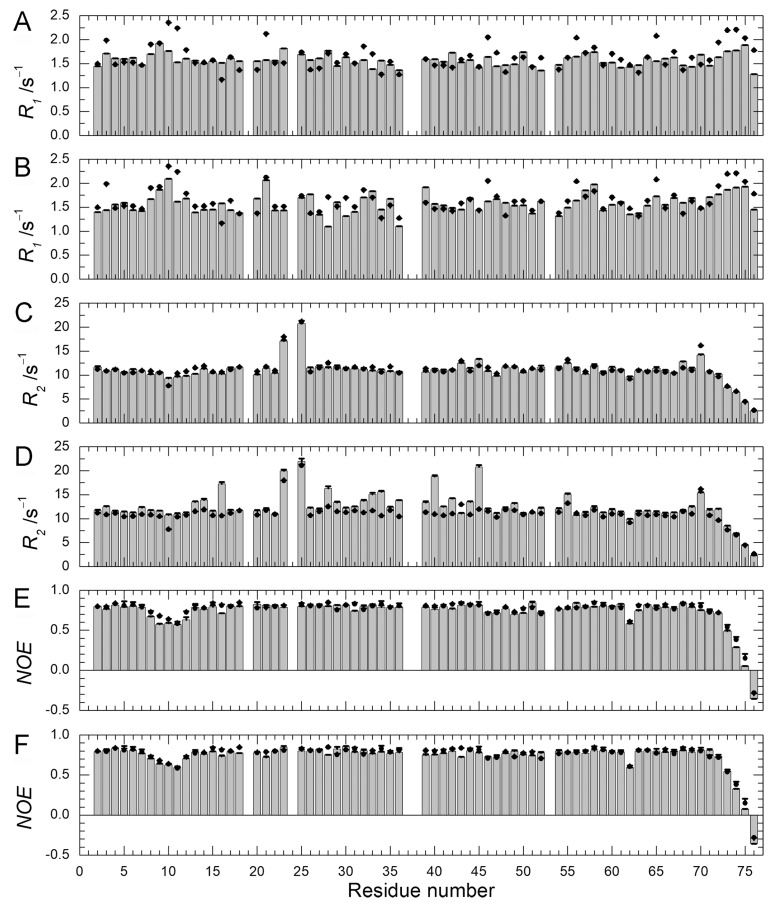
^15^N-spin relaxation dynamics of ubiquitin mutants (E34A and Q41N) (histograms) at 278 K. (**A**) ^15^N-longitudinal relaxation rates, *R*_1_ for E34A; (**B**) ^15^N-longitudinal relaxation rates, *R*_1_ for Q41N; (**C**) ^15^N-transverse relaxation rates, *R*_2_ for E34A; (**D**) ^15^N-transverse relaxation rates, *R*_2_ for Q41N; (**E**) Heteronuclear Overhauser effects, ^15^N-NOE for E34A; (**F**) Heteronuclear Overhauser effects; ^15^N-NOE for Q41N. Data for WT protein are represented by closed circles in each panel. Data for WT and Q41N were obtained from the literature [22] for comparison (Reproduced with permission from American Chemical Society).

We previously performed ^15^N–spin relaxation experiments (^15^N-*R*_1_, *R*_2_, and NOE) on WT protein and Q41N mutant [22]. Here, we performed these experiments on E34A mutant and compared the dynamic backbone motions of the mutants (Q41N and E34A) with those of WT protein. Figure 6 shows ^15^N-*R*_1_ (A & B), *R*_2_ (C & D), and NOE (E & F) values for E34A (A, C, E) and Q41N (B, D, F) at 278 K. ^15^N-*R*_1_ and NOE are sensitive probes of the picosecond-to-nanosecond motions of proteins, whereas ^15^N-*R*_2_ is sensitive to the microsecond-to-millisecond motions. For both mutants, the ^15^N-NOE values were almost identical to those of WT protein.

The ^15^N-*R*_1_ values showed a similar tendency. The similarity of these values between WT and mutants indicates that the picosecond–to–nanosecond motion was not significantly altered by the mutations. In contrast, the remarkable increase in the *R*_2_ values observed at residues 28–45 and 69–72 of Q41N (Figure 6D) shows that the microsecond–to–millisecond motion was amplified at these residue sites in the mutant.

As we previously reported, an increase in *R*_2_ values can be attributed to an increase in exchange contribution to *R*_2_ caused by an increase in the N_2_ population and a decrease in the rate of conformational exchange between N_1_ and N_2_ [18,22]. At 298 K, we observed similar tendencies for ^15^N-*R*_1_, *R*_2_, and NOE (data not shown).We have previously characterized the water-amide proton exchange of the mutants using phase-modulated clean chemical exchange (CLEANEX-PM) experiments. In these experiments, the magnetization transfers from water molecules to amide protons were monitored. Compared to the exchange rates of WT, the Q41N exchange rate was more than 10-times greater at some residues in the α-helix, loop, and β_3_-strand regions [22]. Many of the residues in these regions are involved in the N_1_–N_2_ conformational transition in WT protein. Thus, the observed increases in water-amide proton exchange rates in Q41N strongly supported the reorientation of the α-helix, loop, β_3_-strand, and β_5_-strand regions and the concomitant penetration of water into the hydrophobic cavity, as predicted by high-pressure NMR spectroscopy [18] and high-pressure MD simulations [21] for WT protein. In contrast, in K11A and E34A mutants, no increase in the exchange rates at the corresponding residues was observed, except for residues near the substituted site [22]. Therefore, disruption of the salt bridge between K11 and E34 does not seem to amplify the N_1_–N_2_ fluctuation significantly. The results of ^15^N spin relaxation experiments on the E34A mutant reported here support this hypothesis.

### 2.5. The Free-Energy Landscape

Finally, we discuss the effects of mutations on the relative free-energy levels of the N_1_ and N_2_ states. In the present study, the CD experiments showed that the Δ*G* value for the transition from the folded states (N_1_ and N_2_) to the unfolded state was 33 ± 8 kJ/mol for WT and 17 ± 5 kJ/mol for Q41N, at 298 K. We previously estimated that Δ*G* between N_1_ and N_2_ is 3.4 ± 0.1 kJ/mol for WT and −2.2 ± 0.3 kJ/mol for Q41N, at 298 K [22]. Given these Δ*G* values for WT and Q41N mutants, we estimated the relative energy levels among the N_1_, N_2_, and U states of the proteins. Here, we assumed that the totally unfolded states of both WT and Q41N have the same free energy.

**Figure 7:**
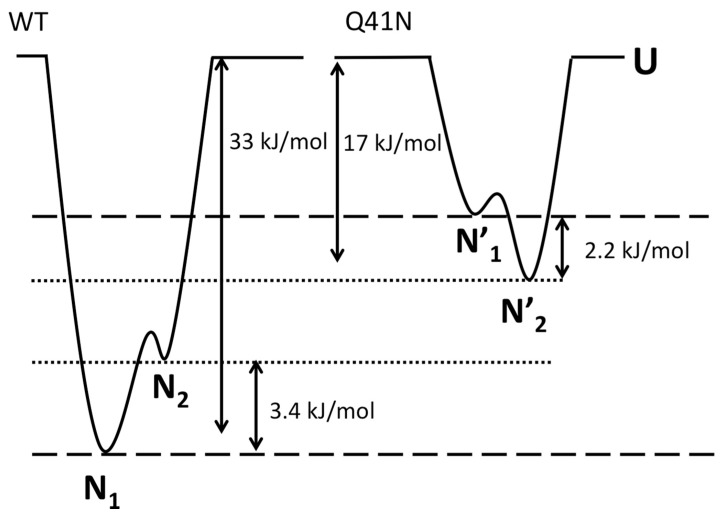
The Gibbs free energy diagrams for the N_1_, N_2_, and unfolded (U) states of WT (**left**) and Q41N (**right**) ubiquitin at 298 K. Δ*G* between N_1_ and N_2_ for WT and Q41N at 298 K were obtained from the literature [22].

Figure 7 shows a sketch of the energy landscape for WT and Q41N. Comparing these free energy diagrams, we found that the Q41N mutation destabilizes the N_1_ state (N_1_→N_1_’) more strongly than N_2_ state (N_2_→N_2_’). Because the N_2_ conformer contains more open and hydrated surfaces at the C-terminal side than the N_1_ conformer [23], we speculate that the enthalpic gains from the hydrogen bond between I36 and N41 in Q41N (N_2_ model) were outweighed by the entropic gains from the hydration of the C-terminal side of the protein. Indeed, CLEANEX-PM experiments on Q41N showed that the hydrated region was expanded at residues 31–41 [23]. These hydration may bring conformational heterogeneity of residues 31–41, namely the gains of conformational entropy. Interestingly, similar hydration-induced conformational changes were observed in the L69S mutant of ubiquitin [29], in which hydrophobic interactions and van der Waals interactions at the C-terminal side of the protein were reduced. In this case, a reduction in noncovalent interactions at this region, caused by mutation, could increase the N_2_ population.

## 3. Materials and Methods

### 3.1. Sample Preparation

Wild-type ubiquitin uniformly labeled with ^13^C/^15^N and ^15^N-labeled ubiquitin mutants (E34A and Q41N) were produced by conventional *Escherichia coli* expression and purified by ion exchange and gel column chromatography. The protein concentration was adjusted to 1–1.5 mM for NMR experiments and to 0.1 mM for circular dichroism experiments, in 20 mM d–Tris-HCl (or Tris-HCl) buffer solution at pH 7.2 and 298 K. For NMR samples, 7% ^2^H_2_O was included in the solution.

### 3.2. Circular Dichroism Measurements

CD spectra of ubiquitin and its mutants (E34A and Q41N) were measured at 215–245 nm wavelength at various concentrations of GdmCl using a J-805 spectropolarimeter (JASCO Co., Tokyo, Japan) at 298 K. CD absorption values at a wavelength of 222 nm were separately measured as an average of 100 scans. Error-bars show root-mean-square-deviation from the average, which were estimated from 100 scans. A quartz cuvette with a path length of 1 mm was used for all measurements. Molar ellipticity, *θ*_222_ per residue at different GdmCl concentrations was calculated from the absorption at 222 nm. *θ_obs_* can be expressed by Equation (1):

(1) $$\theta_{obs}=\frac{\theta_{F}+\theta_{U}\;\exp\left[-\frac{\left(\Delta G_{N\to U}^{0}-m\times c\right)}{RT}\right]}{1+\exp\left[-\frac{\left(\Delta G_{N\to U}^{0}-m\times c\right)}{RT}\right]}$$

where$\;\Delta G_{N\to U}^{0}$ is the Gibbs free energy change for unfolding, *m* is the *m*-value, *c* is the concentration of GdmCl, *R* is the gas constant, and *T* is the absolute temperature. *θ_F_* and *θ_U_* are the molar ellipticities of the folded and unfolded species, respectively. Assuming that *θ_F_* and *θ_U_* were constants (baseline values), *θ_obs_* was fit to Equation (1) with four variables, Δ*G*^0^, *m*, *θ_F_* and *θ_U_*. 

### 3.3. NMR Measurements and Analysis

All NMR experiments were performed on a DRX-600 spectrometer (Bruker BioSpin Co., Fällanden, Switzerland) at a ^1^H frequency of 600.13 MHz. Signal assignments for Q41N were obtained by triple-resonance NMR experiments, as reported previously [22,23]. All HSQC cross-peaks for other mutants were assigned to individual amide groups using ^15^N-edited TOCSY and NOESY measurements, with reference to the assignments of the WT and Q41N proteins. ^1^H chemical shifts were referenced to the methyl signal of DSS, and ^15^N chemical shifts were indirectly referenced to DSS (0 ppm for ^1^H). Data were processed using the software Topspin 1.1 (Bruker BioSpin Co, Fällanden, Switzerland) and NMRPipe [30] and analyzed using the software NMR view [31] and KUJIRA [32].

Two-dimensional H(C)CO and HC(CO) experiments were performed on WT protein at 298 K between pH 1.98 and pH 6.88, at intervals of approximately 0.25 pH (18 different pH values), based on the Bruker HCACO pulse sequence with offsets altered to fit to the Cβ/Cγ shifts [26]. Assuming two extreme chemical shifts corresponding to protonated and unprotonated species, we determined the p*K*_a_ values for aspartate and glutamate by the global fitting of the observed chemical shift data from H (C) CO and HC (CO) spectra to a Henderson-Hasselbalch equation. The details have previously been described [27,28].

^15^N spin-relaxation parameters, ^15^N-*R*_1_, *R*_2_, and NOE were obtained for all mutants at 278 K and 298 K, using previously described pulse sequences [33]. Spin-lattice relaxation measurements and spin-spin relaxation measurements were performed with 10 mixing times (10, 20, 40, 70, 100, 200, 400, 600, 900, and 1200 ms for *R*_1_ and 20, 35, 50, 70, 100, 130, 160, 200, 250, and 280 ms for *R*_2_). Relaxation rates were obtained by fitting the cross-peak intensities to a single-exponential function. ^15^N-NOE measurements were performed with 3-second intervals. Spectra were recorded with and without pre-saturation of the amide proton signals during the 3-second interval. The ^15^N-NOE value represents a ratio of cross-peak intensities with/without pre-saturation, and the value was obtained using the average of two experiments.

## 4. Conclusions

We have determined the p*K*_a_ values for the aspartate and glutamate side chains based on pH-dependent chemical shift changes of Hβ/Hγ, Cβ/Cγ, and C′; these data suggest that most of the salt bridges in the protein do not significantly influence protein stability and structure in solution. In particular, the salt bridge between K11 and E34 does not substantially contribute to the N_1_–N_2_ fluctuations or protein stability. In contrast, the hydrogen bond between I36 and Q41, which is conserved among ubiquitin-family proteins, is crucial for the N_1_–N_2_ fluctuations. Thermodynamic analysis revealed that the larger population of N_2_ in Q41N can be attributed to the relatively greater destabilization of N_1_ than N_2_. A suitable point mutation based on the structure and dynamics of high-energy protein states enabled us to determine the specific interactions controlling a particular motion of ubiquitin. The present strategy is generally applicable to any protein and shows potential for facilitating molecular design for functional dynamics beyond the native state.

## Figures and Tables

**Table 1:** Carboxyl p*K*_a_ values for wild-type ubiquitin.

Residue	p*K*_a_^a^	p*K*_a_^b^
D21	2.98 ± 0.03	3.1
D32	3.68 ± 0.03	3.8
D39	3.52 ± 0.02	3.6
D58	3.47 ± 0.02	3.7
E34	4.16 ± 0.03	4.5
E16	4.07 ± 0.02	3.9
E18	4.26 ± 0.03	4.3
E51	3.69 ± 0.02	3.8
E64	4.26 ± 0.02	4.5

^a^ 20 mM d–Tris HCl buffer at 298 K (7% D_2_O). ^b^ 100 mM KCl at 303 K (10% D_2_O) [27] (Reproduced with permission from American Chemical Society).
